# Associations between the helpfulness of teacher induction programs, teacher self-efficacy, and anticipated first-year teacher retention

**DOI:** 10.3389/fpsyg.2023.1088111

**Published:** 2023-02-23

**Authors:** Xiaotian Han

**Affiliations:** Department of Elementary School Education, School of Primary Education, Shanghai Normal University Tianhua College, Shanghai, China

**Keywords:** teacher induction programs, teacher self-efficacy, teacher retention, primary school teachers, first-year teachers

## Abstract

First-year teachers need help because they are confronted with various challenges and are more likely to leave the profession within a few years. Studies have demonstrated the efficacy of evidence-based teacher induction programs (TIPs) in enhancing the performance of new teachers and promoting positive student outcomes. However, there has been opposition to this assertion, with some suggesting alternative explanations for the observed effects. This study applied Horn et al's high-quality TIP model as the theoretical framework and employed a non-experimental, correlational design to address the research questions by collecting data from 408 first-year primary school teachers in Shanghai. Correlations and multiple regressions were examined in the study. The results revealed the following: (1) the perceptions of the helpfulness of TIP were not found to correlate significantly with teacher self-efficacy; (2) there was a limited negative correlation (*r* = −0.142, *p* < 0.01) between self-efficacy and anticipated retention, suggesting that higher self-efficacy scores were associated with low anticipated retention, contrary to the study's hypothesis; (3) anticipated retention was found to be significantly affected by gender, major, and ratings of TIP. Anticipated retention was found to be significantly affected by gender, major, and ratings of TIP helpfulness. The results, implications, and recommendations are discussed further in the study.

## 1. Introduction

In recent years, supporting and retaining novice teachers has become a critical issue worldwide due to the rising teacher attrition rate. Researchers found several indicators that first-year teachers are confronted with various challenges and are likely to leave the profession within the first 5 years. Some of the challenges include establishing their teaching identity, meeting performance expectations, managing job-related stress and work demands, handling heavy teaching loads and tough competition, navigating relationships with colleagues, administrators, parents, and students, and having a general lack of self-efficacy (Ingersoll, 2012; Headden, 2014; Atteberry et al., 2015; Banville, 2015; Ren, 2016; Zee and Koomen, 2016; Wu, 2018). Even though there is no single solution to address the issue of high turnover rates among first-year teachers, TIPs have been recognized and praised as a possible solution. TIPs are viewed as bridges that facilitate the transition of “student teachers to teach students” (Smith and Ingersoll, 2004, p. 683). Evidence-based TIPs have shown great promise in improving teachers' overall performance and students' outcomes (e.g., Bastain and Marks, 2017; Kwok et al., 2022). TIPs equip novice teachers with strategies for managing stress, cultivate a strong sense of teacher identity, and foster a community of support, thereby enhancing their self-efficacy in areas such as classroom management, student engagement, and instructional delivery. Skeen et al. (2020) examined the changes in the cohort's overall teacher efficacy ratings from their first to their third year of teaching. They used a nine-point scale developed by Tschannen-Moran and Woolfolk Hoy (2001) and demonstrated that the third-year teachers' overall teaching efficacy rating increased by 1.22 points, with their classroom management rating increasing by 1.78 points. However, improving ratings in a particular field does not indicate that the helpfulness of TIP is statistically correlated with teacher performance and positive student outcomes.

Several researchers concluded that TIP has little or no impact on positive student outcomes or teacher retention based on their observations in one- or two-year TIPs (e.g., LoCascio et al., 2016). Moreover, a recent study focused on limited themes and content, highlighting a growing need for further investigation into the content and impact of TIPs (Gibbons and Cobb, 2017; Kraft et al., 2018). In addition, although the relationships between the effectiveness of TIPs, teacher self-efficacy, and their anticipated retention have been discussed, there remain some questions, such as how are these factors correlated? Is there a mediator between these factors? Saffold (2005) writes, “The perception that one's teaching has been successful increases efficacy beliefs, thus raising expectations that future teaching performances will be successful. In contrast, failure, especially if it occurs early in the learning experience, undermines one's sense of efficacy” (p. 1). Moreover, Shearn (2007) identified the effectiveness of TIPs as “the most influential predictor of sense of efficacy” through his study and a detailed description of the sample of 225 first-year teachers (p. V). Other researchers also demonstrated that TIPs could improve teacher efficacy beliefs (Allen, 2014; Dangler, 2016; Alia et al., 2017; Lemon and Garvis, 2017). However, researchers have critiqued the positive relationship between these two variables and indicated no relationship between the types of TIPs and teacher efficacy (Lowrey, 2012). In addition, no empirical study has yet examined the relationship between the helpfulness of these factors associated with teacher self-efficacy and/or first-year teacher retention.

The participants of this study were first-year teachers who were new to public schools in Shanghai (Shanghai Municipal Education Commission, 2013). The criteria for first-year teacher retention in this study were defined as either remaining in the same teaching position in a Shanghai public school or transferring to another public school within Shanghai. This study focused on first-year teachers in Shanghai public primary schools and aimed to address descriptive and correlational research questions.

Research Question 1 (Path a): Is there an association between the helpfulness of teacher induction programs and teacher self-efficacy after controlling for gender, educational level, and major?

Research Question 2 (Path b): Is there an association between teacher self-efficacy and anticipated teacher retention after controlling for perceptions of TIP helpfulness, gender, educational level, and major?

Research Question 3 (Path c): Is there an association between the helpfulness of teacher induction programs and anticipated teacher retention after controlling for gender, educational level, and major?

Research Question 4: Is there an indirect effect of the helpfulness of teacher induction programs on anticipated teacher retention *via* teacher self-efficacy?

## 2. Literature review

### 2.1. Historical development of TIPs in Shanghai

The definitions of TIPs vary in different countries. “The term induction is used to describe the period when teachers have their first teaching experience and adjust to the roles and the responsibilities” (Nielsen et al., 2007, p. 15). TIPs, also known as Beginning Teacher Support and Assistance (BTSA) programs in the United States, are programs aimed at providing new teachers with the skills and knowledge needed to succeed in the classroom. These programs focus on helping novice teachers develop effective teaching strategies and adjust to the demands of the profession. They were also regarded as bridges transforming “student teachers to teach students” (Smith and Ingersoll, 2004, p. 683). TIPs are known as pre-planned, structured, and short-term assistive programs offered in schools for novice teachers. In the United Kingdom, TIPs were called Teacher Induction Schemes (TIS). TISs are focused on addressing various concerns for novice teachers, such as “the structure of their induction into teaching, the traits of their induction supporter, and their development needs… in their future support and development” (Rippon and Martin, 2006, p. 86). Shanghai Municipal Education Commission (2013) views TIPs as the beginning of a teacher's long-term professional development journey in public schools. The main purpose of TIPs is to provide new teachers with support and guidance from experienced mentors in their first year, with the aim of improving teacher quality and effectiveness in classroom instruction and collaboration, ultimately leading to higher retention rates among new teachers (Report on U.S. Department of Education, Institute of Education Sciences, What Works Clearinghouse (2015, July).

Compared to TIPs in the United States and other European countries, which have a long history of supporting first-year teachers, TIPs in Shanghai are relatively new. There are two versions of TIPs in Shanghai: the old version prior to 2012 and the formalized version implemented in 2012 (Chen and An, 2016). TIPs in Shanghai were proposed in 1985, developed in 1999 by the Shanghai Education Commission, implemented in 2001, and reformed in 2012. The initial aim of creating TIPs for Shanghai public schools was to provide on-the-job training for 67,000 unqualified teachers. The previous iteration of inductions indicated that first-year teachers were required to complete over 120 h of in-school training and mentorship during their inaugural year of teaching. However, the specifics regarding the program content, the scope of the training, and the method for organizing the activities were not clearly defined (Chen and An, 2016).

Moreover, since the TIPs varied between schools and districts, first-year teachers received different types of training with different levels of quality, according to many researchers (Chen and An, 2016). To balance the quality of differentiated TIPs, the Shanghai Education Commission declared a new induction program system in 2012. Compared to the old system, the new one involved more resources, such as district training, base schools, and schools where the first-year teachers work. The content of TIPs was standardized and comprised four components: orientation, mentoring, professional growth, and teacher assessment. Upon successful completion of the TIP program, teachers were awarded TIP certificates, which are a partial requirement to renew their teaching credentials (Order No. 55, Shanghai Municipal Education Commission, 2013).

### 2.2. Key components of TIP in Shanghai's teacher public-school education system

Many studies have discussed various components of TIPs that are useful in providing support to first-year teachers. TIPs included seminars and workshops, mentoring, collaboration sessions with colleagues and administrators, program assessment, and teacher assessment (Clark, 2012; Gaikhorst et al., 2015). Ingersoll (2004) identified that the factors of TIP activities were mentoring, newly qualified teacher in-service activities, class observations, provision of curriculum guides, instructional materials/resources support, ongoing new teacher meetings, and collaborations between colleagues. Meristo and Eisenschmidt (2012) claimed that the content of TIPs includes providing support to new teachers, mentoring and analysis, and implementation. Banville (2015) emphasized the importance of understanding the teaching context, designing a flexible instructional program, building a positive classroom environment, establishing a foundational skillset, and establishing a professional identity. Lofstrom and Eisenschmidt (2009) pointed out that TIPs should incorporate a combination of general studies, such as cultural and social competencies, specialized studies that involve the integration of human beings with their communities, as well as pedagogical strategies.

Horn et al's (2002) model was a comprehensive tool to synthesize high-quality TIPs and served as the basis for evaluation. Moreover, the components of TIPs in Shanghai highly match the Horn et al. (2002) model. The four components were orientation, mentoring, professional growth, and teacher assessment. The orientations (5 days) of TIPs in Shanghai are in the summer before the new school year. The content of district orientation includes introducing educational policies and laws, professional career development, and morals (Shanghai Educational Municipal Commission, 2017). The content of school orientation includes meeting on-site administrators, teachers, and staff members, declaring mentors and mentees, and discussing current educational issues and tasks (Ren, 2016).

The second component of TIP is mentoring. Horn et al's (2002) research defined mentoring as “one in which the administration has a mentoring program in place with specific guidelines, programs are funded, mentors are compensated in some way, and there are specific expectations and policies regarding the mentoring process” (p. 24). Mentors are generally appointed by school administrators or universities and participate in supervision (Lofstrom and Eisenschmidt, 2009). In Shanghai TIPs, mentors are experienced teachers selected by on-site school principals, providing first-year teachers with “new apprentices with guidance on course preparation, coursework evaluation, and organization of student activities and so on” (Zhang et al., 2016, p. 14). They role play as buddies, trainers, listeners, and supervisors to provide support to teachers who are new to the school, the grades, and the subjects (Nielsen et al., 2007; Ingersoll, 2012). In Shanghai TIPs, the role of mentors is more like that of an instructional coach, handing over his or her experience to the first-year teachers (Shanghai Municipal Education Commission, 2012; Chen and An, 2016). According to the Shanghai TIP Handbook (Edited in 2017), mentors have the following duties: (1) Following Shanghai's formalized teacher induction program content and request, a mentor has to follow new teachers' professional growth in the school year in the four sections of professional identities and ethics, instructional strategies, classroom management, moral education, and research methodology and career development; (2) a mentor has to implement and record mentoring activities (including objectives, procedures, and evaluations); (3) a mentor has to help a mentee in understanding the curriculum and standards, writing lesson plans, providing feedback and suggestions on instructional strategies and class management, observing classes and offering feedback, and doing formative evaluations periodically; (4) a mentor has to follow, implement, and fill out the Shanghai TIP Handbook; and (5) a mentor should have no problem with supervision by the school, the district, and the Shanghai Educational Commission. A mentor should consider participant-mentor-mentee collaborations for at least 1 h per week. Therefore, mentors must possess strong teaching abilities and professional expertise in areas such as motivation, listening, and reflection (Harrison et al., 2019).

The third component of the Shanghai TIP is professional development. Horn et al. (2002) defined professional development as providing opportunities for first-year teachers to obtain additional knowledge and skills and develop attitudes necessary for successful teaching. In Shanghai TIPs, first-year teachers spend 2 days with mentors at the school site and 3 days engaged in professional development. The professional development comprises the following: (1) half a day per week for district-level professional development activities and (2) 2.5 days per week at the base school (Chen and An, 2016). These activities may include workshops/seminars with experts and professors from local universities, case studies, peer observations, group discussions, and collaborations among first-year teachers. The professional development topics cover four sections: teacher identities and ethics, instructional strategies and class practices, class management, moral education, and research techniques (Shanghai Educational Municipal Commission, 2017).

The last section of TIP is evaluation. Horn et al. (2002) believed that the purpose of teachers was to assess first-year teachers' strengths and weaknesses through self-assessment and evaluations by mentors, school administrators, and district representatives. Moreover, Shanghai TIP evaluations are regarded as an essential tool in determining whether first-year teachers are able to stay in their profession. Ren (2016) also discussed that the reasons for teacher evaluations are as follows: (1) evaluating the implementation of the program and (2) evaluating teachers' achievements. In Shanghai TIPs, first-year teachers are evaluated through self-assessment, their mentors (in the base school and the on-site school), administrators (in the base school and the on-site school), and district representatives through program activities, attendance, and how close they came to completing the program. The detailed evaluation forms are attached below. In the attached evaluation form, the first-year teacher fills in background information and reports on her 1-year TIP experience. The base school mentor (subject mentor) writes comments and gives scores according to the scoring criteria. If the first-year teacher works as a classroom teacher, the teacher mentor also needs to provide comments. Similar to the base school mentor(s), an on-site school mentor should provide comments and give an overall evaluation. Once new teachers and mentors fill out the forms, the district representatives determine whether the first-year teacher completed TIP at the level of “exemplary,” “fair,” “at standard,” or “below standard.”

### 2.3. Teacher self-efficacy theory

Self-efficacy is an individual's belief in their ability to perform a particular action successfully (Bandura, 1997, 2006). It means “can do” rather than “will do.” In other words, self-efficacy is what humans believe they are capable of doing. Self-efficacy should be distinguished from other similar constructs, such as self-esteem and locus of control. Efficacy beliefs influence human attitudes and anticipated actions. Attitudes include thinking through a problem erratically, strategically, optimistically, or pessimistically (Bandura, 2006). Anticipated actions could involve determining whether human beings choose to pursue a challenging task, how long and how much effort they will put in, and how much stress and depression they can cope with when they experience difficulties. “Weak efficacy beliefs are easily negated by disconfirming exercises, whereas people who have a tenacious belief in their capabilities will persevere in their efforts despite innumerable difficulties and obstacles” (Bandura, 2006, p. 314). However, if a person has a strong sense of personal efficacy, he or she is more likely to successfully perform the chosen activity.

Based on Bandura's social cognitive theory and its effects on human behaviors, teacher efficacy is defined as “beliefs in one's capabilities to organize and execute the courses of action required to produce given attainments” (Bandura, 1995, p. 3). The definition has been accepted by researchers. Other researchers also provided alternative definitions for teacher efficacy. Tatar and Buldur (2013) defined teacher efficacy as “one's capabilities to organize and supervise the course of action needed for managing prospective situations” (p. 453). Tschannen-Moran and Woolfolk Hoy (2001) considered teacher efficacy as a judgment of a teacher's capabilities “to bring about desired outcomes of student engagement and learning, even among those students who may be difficult or unmotivated” (p. 783). Berg and Smith (2018) emphasized teacher efficacy as teachers' beliefs about their ability to plan, organize, and deliver instructions to attain given educational goals. These definitions commonly underscore teacher efficacy, as teachers' beliefs are correlated with professional development and positive student outcomes.

In education, teacher self-efficacy affects student learning outcomes and the quality of instructions provided by the teacher. First, teacher efficacy affects the extent to which they can positively impact student performance and learning outcomes, even for students who face difficulties with learning (Skaalvik and Skaalvik, 2019). Liu et al. (2005) investigated 109 teachers and 3,066 students in primary schools in China and found that teacher efficacy is associated with student learning attitudes. Moreover, teachers with high levels of self-efficacy generally exhibit positive attitudes toward teaching and goal setting, show strong planning and organizational skills, employ differentiated instruction strategies, and create a positive and supportive instructional environment (Stephanou and Oikonomou, 2018). Yin et al. (2013) discovered through a survey of 1,646 primary and secondary school teachers from six provinces in China that teacher efficacy has a mediating effect on the level of trust among colleagues and teacher empowerment. The study design of Yin et al. (2013) identified trust in colleagues among teachers as an independent variable, teacher empowerment as a dependent variable, and teacher self-efficacy as the mediating factor. The research results showed the following: (1) teachers' perception of trust in colleagues significantly impacts their sense of empowerment in the school; (2) when controlling for teacher efficacy, the study found that teacher efficacy has a significant effect on teacher empowerment in schools; and (3) the results also revealed that teacher efficacy has a complete mediating effect on the relationship between teachers' trust in colleagues and teacher empowerment in schools. This highlights the crucial role that teacher efficacy plays in professional development and teacher retention rates.

### 2.4. Challenges regarding anticipated teacher retention

First-year teachers have high motivation for teaching and learning in general. They desire to work with children and adolescents, stimulating their learning/teaching attitudes, expectations, and engagement in the first year (Watt and Richardson, 2008). However, first-year teachers also face various extrinsic challenges. Low teacher efficacy beliefs contribute to teacher attrition (Schaefer et al., 2012). Several studies showed that teachers' sense of self-efficacy predicts difficulties in adapting to the teaching context and an increased likelihood of attrition. Klassen and Chiu (2010) conducted a study with a sample of 1,430 practicing K-6 teachers and found the following: (1) higher levels of teacher efficacy in classroom management and instructional strategies correspond with higher job satisfaction among teachers and (2) increased job-related stress (i.e., classroom stress and workload stress) is linked to lower teacher efficacy. Babaei and Abednia (2016) also found a positive correlation between teacher reflectiveness and teacher efficacy in a study of 225 Iranian English as a foreign language teachers. Savaş et al. (2014) studied 163 primary and secondary teachers and found that teacher efficacy was significantly and negatively associated with the likelihood of burnout. Yost (2006) discussed the potential cause-effect relationship between teacher efficacy and retention. He also pointed out that the opportunity for professional development is the key factor determining teacher efficacy. Canrinus et al. (2012) proved that teachers with greater classroom self-efficacy have a greater sense of their professional identity (i.e., commitment, motivation, and job satisfaction); these are findings from the study of 1,214 Dutch teachers as participants. In addition, the association between teacher efficacy and the likelihood of job burnout was evident in a study conducted in China (Yu, 2015).

In addition to the general first-year teachers' challenges that were listed, first-year teachers in Shanghai public primary schools face additional challenges. First, newly qualified teachers may not have enough training because teacher preparation programs are not mandatory in Shanghai's public primary schools. A primary school teacher is required to have a bachelor's degree (4-year college/university degree) or a higher degree and a teaching credential. Teachers in Shanghai's public primary schools are all specialists rather than all-subject teachers as they are in other countries. Based on their college majors, teachers can be categorized into three groups: general education, core course majors (i.e., Chinese, Math, and English), and other course majors (i.e., science, arts, and physical education). Teachers who major in education undergo teacher training in a college setting, resulting in automatic certification (Xia, 2018). However, the standards for teacher training programs vary across different universities. For teachers whose majors were other than education (core course majors and other majors) and who were willing to train to teach, they did not experience any teaching practice but focused on all subject-based courses in their universities. They must take a teaching credential test (Xia, 2018). The credential test is a law and subject knowledge-based written and oral test. It does not require class practice hours. Therefore, both groups of first-year teachers may lack real classroom teaching experience when hired.

Besides, first-year teachers are expected to build positive and stable relationships with colleagues, administrators, parents, and students (Ren, 2016). However, they always feel powerless and isolated in their first years (Zhao, 2003). Cao and Zhou (2007) pointed out that dealing with the student–teacher relationships is the greatest challenge first-year teachers face. Knowing students well and having positive relationships with them are related to course design, planning, and organization, motivating students' interests, and delivering differentiated instructions. However, first-year teachers are not confident in building the teacher–student relationships. In addition, Shanghai public school teachers face high competition and a heavy workload. They are expected to show higher student academic achievements *via* standardized exams and a series of government interventions than teachers in some other countries (Shanghai Educational Municipal Commission, 2017). To improve students' academic scores, they must bear heavy workloads; their average working time is 9.16 h per day without extra-time payment (Wu, 2018). Considering newly qualified teachers' internal motivation and the external challenges they face, first-year teachers are overwhelmed by dealing with these imbalances. If there is an imbalance, a low job retention rate is noticeable.

## 3. Research design

The study employed a non-experimental, correlational design and used teacher survey responses to address the research questions. The teachers provided information about their backgrounds, their perceptions of how helpful they found the TIPs to be, their sense of teaching efficacy, and their plans regarding remaining in teaching. The study, therefore, did not involve longitudinal data collection, although that would more easily lend itself to causal inferences. Recognizing that interpretation must proceed cautiously, the study's logic is that the teachers' responses having to do with prior, current, and future events allow exploration of the possible impact TIP components may have on teacher retention.

### 3.1. Population and samples

The target population was first-year teachers in Shanghai public primary schools. The selection criteria were that participants had a bachelor's degree or higher along with a teaching credential and were in their first year of teaching in a public primary school in Shanghai. Due to practical constraints, a convenience sample was utilized in this study.

We considered the following to determine how many teachers to invite to participate in the study. After dummy-coding the control variables (gender, education level, and major), there were five predictors along with two main variables (the helpfulness of teacher induction programs and teacher self-efficacy) for a total of seven predictors in the most complex model tested. The software G^*^Power 3.1.9.3 was utilized by specifying the alpha level to be 0.05 and the desired power to be 0.80, and one predictor was tested for the increase in R squared estimate. Assuming the effect size was small, the needed sample size would be 395. Assuming a 70% response rate, we needed to recruit at least 564 teachers for the study. The statistical analyses would have a higher power than.80 if the effect was larger or the response rate was higher.

### 3.2. Instrumentations

The data for this non-experimental study was collected through a web-based survey (Survey Monkey). The contents of the survey included four sections: (a) demographic information (i.e., gender, education level, and majors); (b) the perceptions of the helpfulness of the TIP scale (on orientation, mentoring, professional development, and teacher evaluations); (c) the teacher self-efficacy scale (for student engagement, for instructional strategies, and for classroom management); and (d) anticipated first-year teacher retention.

In this study, three instruments were used for data collection and analysis, addressing the research questions. The instruments measuring teachers' perceptions regarding the helpfulness of TIPs were developed by the author. This scale is based on the conceptual framework of high-quality induction programs offered by Horn et al. (2002). It aimed to assess how helpful the first-year teachers perceived the TIP as well as each component (orientation, mentoring, professional development, and evaluation). The scale utilized a 5-point response system for each item, ranging from 0 (not at all helpful) to 4 (very helpful). Higher scores indicated a stronger perception of the TIP being beneficial. The reliability and validity of the responses were investigated using the study data itself and are reported in the results. Prior to its use in the present study, the survey would be piloted with a handful of teachers who had participated in a TIP in Shanghai in recent years.

The tool for measuring teacher self-efficacy was the short form of the Teacher Sense of Efficacy Scale developed by Tschannen-Moran and Woolfolk Hoy (2001). A shorter 12-item form was developed and later translated into Mandarin by Hsin-Chieh Wu, a student of Woolfolk Hoy. Permission to use the Teachers' Sense of Efficacy Scale and the Mandarin version of it is provided in advance. The Teachers' Sense of Efficacy Scale generates three subscale scores: efficacy for instructional strategies (item 5, item 9, item 10, and item 12), efficacy for classroom management (item 1, item 3, item 6, and item 8), and efficacy for student engagement (item 2, item 4, item 7, and item 11). Efficacy for instructional strategies tests whether teachers believe that they are able to provide a variety of assessment strategies, provide an alternative explanation or example when students are confused, ask good questions of students, and implement alternative strategies in the classroom. Efficacy for classroom management explores teachers' beliefs about controlling students' disruptive behavior, helping children to follow classroom rules, and establishing a classroom management system. Efficacy for student engagement concerns teachers' beliefs in their ability to motivate students to learn and do well in school. The scale utilizes a nine-point response system for each item, ranging from 1 (no influence) to 9 (a great deal of influence). The internal consistency and reliability of the 12-item English version have been reported in the study by Tschannen-Moran and Woolfolk Hoy (2001).

The overall reliability for this 12-item scale was 0.90. The reliability scores for the teacher self-efficacy subscales were 0.86 for instructional strategies, 0.86 for classroom management, and 0.81 for class engagement. Information regarding the reliability and validity of the Mandarin version was provided by its author.

The teacher retention scale, developed by the author, was used to assess a teacher's consideration of various career options: (1) staying in the same teaching position, (2) relocating to a different public primary school, (3) relocating to a private school, (4) relocating to a private institution other than private schools, and (5) changing to a different profession. The scale used a 5-point response option for each item, with anchors at 1 (strongly disagree), 2 (disagree), 3 (undecided), 4 (agree), and 5 (strongly agree). In this study, the anticipated first-year teacher retention focuses on teachers staying in the same teaching position at a Shanghai public school or relocating to a different public school. Therefore, items 3, 4, and 5 needed to be scored in reverse. After reversing the scores for these items, a higher score indicates a greater likelihood that the first-year teacher has the intention to continue as a public primary school teacher in Shanghai. The reliability and validity of the study were investigated using the collected data, and the results were reported.

### 3.3. Demographics

In addition to the aforementioned three scales, the survey included a section on the demographic background. Questions such as the participant's age, gender, education level, major, subject, salary, and workload were asked. The data collected were used to describe the participants, and some of this information served as control variables in the main analysis.

This study applied three control variables: gender, college major, and degree level. Research studies demonstrated the influences of gender, major, and degree levels on teacher efficacy and anticipated retention (Klassen and Chiu, 2010; Struyven and Vanthournout, 2014; Wu, 2018). Female teachers reported having higher levels of teacher efficacy than male teachers (Klassen and Chiu, 2010). Ding (2010) also found similar results when measuring teacher self-efficacy in China. Moreover, research studies revealed that female teachers are more likely to stay in teaching positions than male teachers (Ding, 2010; Struyven and Vanthournout, 2014). The developers of the Teacher Self-Efficacy Scale (Tschannen-Moran and Woolfolk Hoy, 2001) included major and degree levels in their analysis. Ding (2010) and Wu (2018) also used both variables in exploring teacher self-efficacy, teacher professional development, job satisfaction, and anticipated job retention in China and Shanghai, respectively.

Not all categories were considered individually for each of the control variables. For example, there are two gender categories (man and woman); thus, gender was one dummy variable (e.g., women coded “1” and men coded “0”). There were two degree level categories (bachelor's and above bachelor's); thus, “graduate” (master's and doctorate) was coded “1” and bachelor was coded “0.” Moreover, the majors were categorized into two groups: education majors were assigned a code of “0,” while non-education majors (including Chinese, English, mathematics, science, music, the arts, and others) were assigned a code of “1.”

### 3.4. Assumptions

It is assumed that TIPs, as a form of teacher training, are carried out according to the government's specifications. In other words, TIPs in Shanghai include relevant action plans based on Horn et al's (2002) theory; TIP providers in Shanghai effectively deliver the TIPs as intended; and TIP participants receive the relevant designed TIP “active ingredients” and put new skills and behaviors into practice (Bellg et al., 2004). Moreover, Horn et al's (2002) conceptual model of the teacher induction program is assumed to fit this study.

## 4. Results

The study posits that the perceived helpfulness of TIPs affects teacher self-efficacy, which in turn impacts their anticipation of staying in teaching. Hence, the level of perceived helpfulness of the TIPs is considered the independent variable, teacher self-efficacy is the mediating factor, and anticipation of teacher retention is the dependent variable.

### 4.1. Descriptive statistics

According to the demographic information regarding the 408 respondents, nearly 70% of the participants' ages were 23–25 years. The percentages of women and men were nearly 85% and 15%, respectively. The percentage of participants who held bachelor's and master's degrees was 91.4% and 8.6%, respectively. No one had a doctorate degree. Regarding majors, about 20% of participants were in education, 40% were in core course majors (Chinese literature and arts, applied mathematics, or English), and 40% were in elective course majors (sciences, music/arts, or others). Nearly half of the participants taught core courses (Chinese literature and arts, applied mathematics, or English), and the other half taught elective courses (music/arts, physical education, science, technology, or others). The primary salary range was RMB¥ 5,001–7,500 monthly. Moreover, about 60% of participants reported that their average teaching workload with students present was 21–25 class periods per week (where one class lasts 35 mins).

Cronbach's alpha was calculated to estimate the scale's internal consistency and reliability. The overall reliability for these 4-item perceptions of helpfulness regarding TIPs was 0.882. Higher scores indicated that the participants felt that TIPs worked for them. Across the four aspects of TIP helpfulness (orientation, mentoring, professional development, and teacher evaluation), the mean of 3.34 indicated that teachers, on average, viewed the TIP as “helpful.” Teachers perceived the TIPs to be most helpful in terms of mentoring and least helpful in terms of teacher evaluation.

The overall reliability of the 12-item teacher self-efficacy scale was 0.938. The reliability for the teacher self-efficacy subscales was 0.888 for instructional strategies, 0.862 for classroom management, and 0.822 for class engagement. Higher scores on this teacher efficacy measure indicate a higher level of confidence in teachers' abilities to achieve desired student outcomes.

The results revealed that the three elements of teacher efficacy had a mean of 79.78 (corresponding to an item average of 6.65, which corresponds to “quite a bit”). The scores for each subscale are highly similar and correspond to teachers reporting that they feel “quite a bit” of efficacy with respect to instructional strategies, classroom management, and student engagement.

On the scale of anticipated first-year teacher retention, after reverse-scoring items 3, 4, and 5, Cronbach's alpha was calculated. The result showed that Item #2 was problematic, as it lowered the reliability to only 0.530. When removed, the four-item scale reached an acceptable level of reliability (a = 0.781). Thus, the remaining analyses were based on the four-item scale (without item #2). Thus, the remaining analyses were based on the 4-item scale (without item #2). Averaging the four items, we found that the mean of anticipated teacher retention was 4.16, which suggests that, overall, the first-year teachers agreed with statements reflecting intentions to stay (and, relatedly, disagreed with statements reflecting an intention to leave) teaching in a Shanghai public primary school.

Based on the results in Table 1, TIP helpfulness (*r* = 0.310) and teacher self-efficacy (*r* = −0.142) were both significantly correlated to anticipated teacher retention. However, TIP helpfulness and teacher self-efficacy (*r* = −0.079) were not statistically correlated. As for control variables, female first-year teachers reported stronger levels of agreement with their plans to stay in the teaching position in Shanghai public schools than men (*r* = 0.117). Teachers with advanced degrees had higher levels of teacher self-efficacy (*r* = 0.108). Teachers who did not complete an education major deemed the Shanghai TIP to be more helpful (*r* = 0.161) and were more likely to stay in public primary schools (*r* = 0.159). However, they experienced relatively lower teacher self-efficacy (*r* = −0.099).

**Table 1:** Descriptive statistics and Pearson correlations between key variables in the regression models with control variables.

**Variables**	**Correlations**				
	**2**	**3**	**4**	**5**	**6**
1. Gender (1 = female)	−0.650	−0.540	0.047	−0.015	0.117^*^
2. Degree (1 ≥ Bachelor's)		−0.054	−0.072	0.108^*^	−0.071
3. Major (1 = non-education)			0.161^**^	−0.099^*^	0.159^**^
4. TIP helpfulness				−0.079	0.310^**^
5. Teacher self-efficacy					−0.142^**^
6. Anticipated teacher retention					

* *p* < 0.05;

** *p* < 0.01.

### 4.2. Research Question 1: Relationship between TIP helpfulness and teacher self-efficacy

Research Question 1 (RQ1) examines the association between TIP helpfulness and teacher self-efficacy after controlling for gender, level of education, and major. It was designed to investigate the influence of the helpfulness of TIP on teacher self-efficacy after controlling for gender, level of education, and major.

The overall teacher self-efficacy scores were regressed on the total rating they gave regarding the helpfulness of the TIP across the four components (orientation, mentoring, training, and evaluation) in which they participated (see Table 2). The full model was statistically significant, *F*_(4, 403)_ = 2.453, *p* = 0.045, with the level of education being the only predictor to account for a statistically significant proportion of unique variation in teacher self-efficacy. Those with education higher than a bachelor's degree had higher levels of self-efficacy (*p* = 0.047). After controlling for gender, level of education, and major, teacher reports regarding TIP helpfulness explained < 1% additional variance, *F*_(1, 403)_ = 1.310, *p* = 0.253, Δ*R*^2^ = 0.003 and were not statistically significant. Thus, for the final research question regarding teacher self-efficacy as a mediating variable, the condition of path “a” being statistically significant was unmet.

**Table 2:** A path analysis of helpfulness of TIPs, teacher self-efficacy, and anticipated first-year teacher retention.

	***b***	***SE_*b*_***	**β**	***t***	***p***
**Control variables**					
Gender (0 = Male)					
Female (1 = Female)	−0.315	1.437	−0.011	−0.219	0.827
Level of education (0 = Bachelor's)					
Graduate degree (1 = Graduate)	3.667	1.843	0.098	1.990	0.047^*^
Major (0 = Education)					
Not education major (1 = NotEd)	−2.171	1.274	−0.085	−0.1.705	0.089
**Predictor variables**					
Helpfulness of TIPs (Path a)	−0.793	0.693	−0.057	−1.145	0.253
Gender (0 = Male)					
Female (1 = Female)	0.165	0.072	0.106	2.280	0.023^*^
Level of education (0 = Bachelor's)					
Graduate degree (1 = Graduate)	−0.054	0.093	−0.027	−0.576	0.565
Major (0 = Education)					
Not education major (1 = NotEd)	0.146	0.064	0.108	2.273	0.024^*^
**Predictor variable**					
Teacher self-efficacy (Path b)	−0.006	0.003	−0.105	−2.242	0.026^*^
Gender (0 = Male)					
Female (1 = Female)	0.167	0.073	0.108	2.293	0.022^*^
Level of education (0 = Bachelor's)					
Graduate degree (1 = Graduate)	−0.074	0.093	−0.037	−0.797	0.426
Major (0 = Education)					
Not education major (1 = NotEd)	0.159	0.064	0.117	2.459	0.014^*^
**Predictor variable**					
Helpfulness of TIPs (Path “c”)	0.210	0.035	0.284	5.982	<0.001^**^

* *p* < 0.05;

** *p* < 0.01.

### 4.3. Research Question 2: Relationship between teacher self-efficacy and anticipated teacher retention

Research Question 2 (RQ2) is designed to examine the association between teacher self-efficacy and anticipated teacher retention after controlling for gender, level of education, and major, as well as teacher perceptions of TIP helpfulness (see Table 2). The full model was statistically significant, *F*_(5, 402)_ = 12.305, *p* < 0.001, with gender, major, teacher self-efficacy, and TIP helpfulness accounting for a statistically significant proportion of unique variation in anticipated retention. The scores in each subcategory are consistent, indicating that teachers have a strong sense of self-efficacy when it comes to implementing instructional strategies, managing the classroom, and engaging students. However, only 1.1% of the 8.9% additional explained variation was due to teacher self-efficacy, *F*_(1, 402)_ = 5.025, *p* = 0.026, Δ*R*^2^ = 0.011. Women agreed more strongly than men that they anticipated continuing their careers as teachers in Shanghai public schools. Those who did not major in education also agreed more strongly than those who did. Individuals who have a higher perception of the helpfulness of TIP (Teacher Induction Program) are more likely to remain in teaching, even when accounting for their level of self-efficacy (i.e., path c' in the mediation model is significant).

Moreover, in directly addressing RQ2, teacher self-efficacy (i.e., path b in the mediation model) is found to be statistically significantly related to anticipated teacher retention. However, the negative coefficient implies that, for each teacher, self-efficacy would increase by a value of one point in first-year teachers in Shanghai public primary schools, and the dependent variable, anticipated teacher retention, would decrease by 0.006 points (*b* = −0.006, *p* = 0.026). The results suggest that first-year teachers who feel greater levels of confidence generally agree less strongly, with items suggesting they anticipate remaining teachers in Shanghai public primary schools. The effect of teacher self-efficacy, however, is small, accounting for only 1.1% of the variation in anticipated retention.

### 4.4. Research Question 3: Relationship between TIP helpfulness and anticipated teacher retention

Research Question 3 (RQ3) examines the association between TIP helpfulness and anticipated teacher retention after controlling for gender, level of education, and major (see Tables 2, 3). The overall anticipated teacher retention scores in Shanghai public primary schools were regressed on the total rating they gave regarding the helpfulness of the TIP across the four components (orientation, mentoring, training, and evaluation) in which they participated. The full model was statistically significant, *F*_(4, 403)_ = 13.986, *p* < 0.001, with gender, major, and TIP helpfulness ratings all accounting for statistically significant proportions of unique variation in anticipated retention. Women agreed more strongly than men, with items measuring anticipated retention, as did those who were not education majors compared to those who did major in education. Directly addressing RQ3, after controlling for gender, level of education and major, teacher reports regarding TIP helpfulness explained 7.8% additional variance, *F*_(1, 403)_ = 35.779, *p* < 0.001, Δ*R*^2^ = 0.078, and was statistically significant and is considered to have a medium effect. When the TIP helpfulness rating increased by a value of one point, the anticipated teacher retention increased by 0.210 points (*b* = 0.210, *p* < 0.001). Thus, for the final research question, regarding teacher self-efficacy as a mediating variable, the condition of path “c” being statistically significant (when the mediator variable was not in the model) was met.

**Table 3:** Mediation test.

	**Effect**	**SE**	***t***	***P***	**LLCI**	**ULCI**
Total effect	0.1624	0.0269	6.0302	0.0000	0.1094	0.2153
Direct effect	0.1517	0.0266	5.9049	0.0000	0.1048	0.2094
Indirect effect (teacher self-efficacy)	0.0052	0.0087	/	/	−0.0090	0.0262

### 4.5. Research Question 4: Mediation

**Figure 1:**
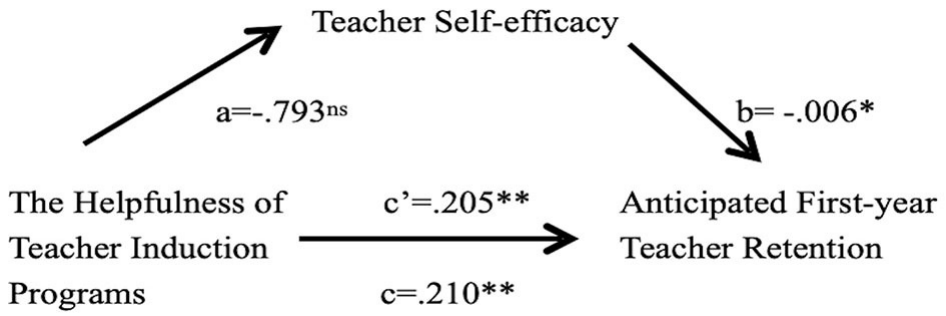
Path diagram with unstandardized coefficients. *p < 0.05; **p < 0.01; ns means not significant.

**Figure 2:**
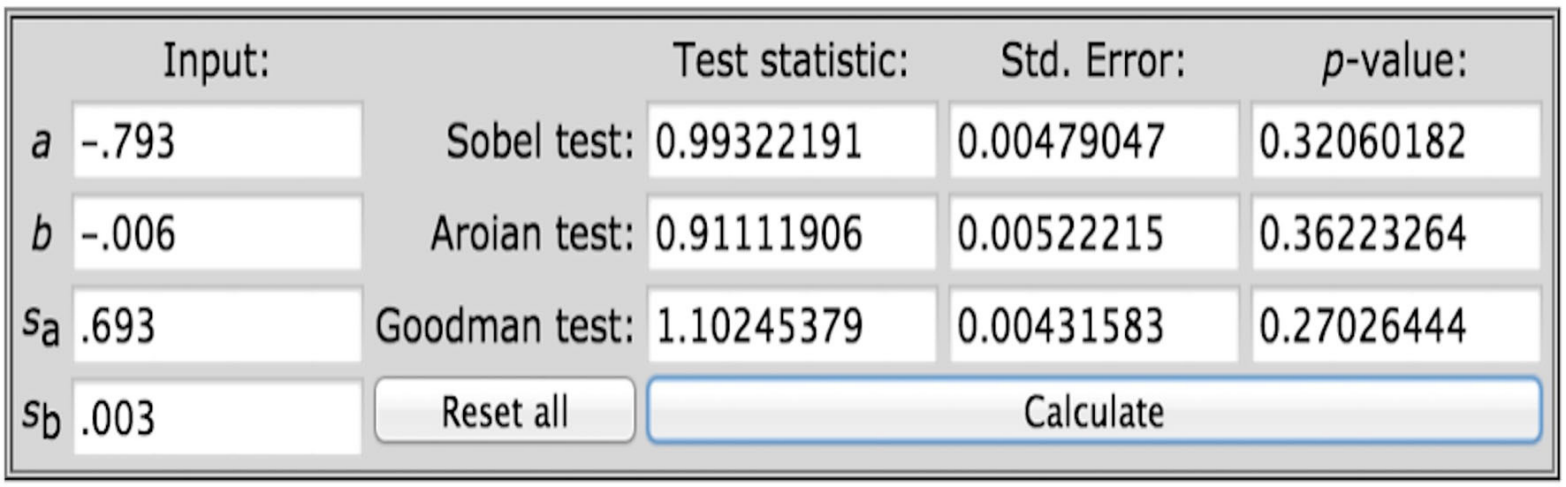
Sobel test to examine mediator.

Research Question 4 (RQ4) aimed to examine whether there is an indirect effect on the helpfulness of TIP and anticipated teacher retention *via* teacher self-efficacy. To answer RQ4, bootstrap analysis with 5,000 random samples was conducted to further explore the mediating role of teacher self-efficacy between the helpfulness of TIPs and anticipated first-year teacher retention. The results revealed that the indirect relationship between the helpfulness of TIPs and anticipated first-year teacher retention *via* teacher self-efficacy was not statistically significant [β = 0.0052, 95% CI = (−0.0090, 0.0262)] (Table 3). Since the result of the bootstrap analysis did not meet the standards of Savaş, Bozgeyik, and Eser (2014) mediation test, there was no indirect effect on the helpfulness of TIP and anticipated teacher retention *via* teacher self-efficacy (Figure 1). In other words, teacher self-efficacy is not a mediator between the helpfulness of TIP and anticipated teacher retention among first-year teachers in Shanghai public primary schools. To further reinforce the conclusion of no mediating effect, a Sobel test was conducted using unstandardized coefficients and their standard errors to examine the indirect impact of the perception of helpfulness on expected teacher retention. Unstandardized coefficients were referred to in Timothy Z. Keith's Multiple Regression and Beyond (2nd edition) (Keith, 2015). It states that “the unstandardized regression coefficients can provide an estimate of the likely change in the dependent variable for each 1-unit change in the independent variable (controlling for the other variables in the regression)” (p. 183). The Sobel Test indicates the result is not statistically significant (*z* = 0.993, *p* = 0.321), which indicates there is no indirect effect of the helpfulness of TIP on anticipated teacher retention *via* teacher self-efficacy (see Figure 2).

## 5. Discussion

RQ 1 verified that the overall TIP helpfulness rating was not a significant predictor of teacher self-efficacy after controlling for gender, educational level, and major. The helpfulness of TIP accounted for less than one percent of the additional variance in teacher self-efficacy. Even though there are few research studies discussing the relationship between TIP helpfulness and teacher self-efficacy in Shanghai or China, the results of this study are inconsistent with the prior literature in other countries and areas. For example, Henry (2016) surveyed 124 new teachers in their first, second, or third year of the induction programs in urban schools and provided Pearson correlation coefficients showing a statistically significant direct relationship between induction effectiveness and teacher self-efficacy. However, Henry's (2016) study defined induction effectiveness in terms of five challenges that new teachers face: planning, handling discipline, communicating with parents, and implementing school district initiatives.

Unlike this study, which defined the TIP using its main activities, Munshi (2018) studied the relationship between teacher induction programs and teacher efficacy by interviewing seven novice teachers, and the findings suggested that mentoring and professional development are two key components in induction programs that “support their [novice teachers'] growing sense of self-efficacy as professionals” (Abstract). However, Munshi's (2018) analysis of each induction component's helpfulness instead of looking at the overall induction program as a predictor of teacher self-efficacy.

In addition, based on the author's perspective and experience during the study, three main reasons may account for this discrepancy. First, some components of the TIPs in Shanghai may have statistically significant effects on teacher self-efficacy among first-year teachers. However, because this study investigates the effect of the helpfulness of TIPs on teacher self-efficacy, these potential relationships may be masked. Second, first-year teachers in Shanghai public primary schools recognized the overall helpfulness of induction programs as a whole and identified that TIP experiences enrich their educational philosophies and theorems (Ding, 2010). However, the current TIPS in Shanghai seem to include less practical learning, leaving the gap open rather than allowing the theorems to be translated into real-world practice. In addition, the self-efficacy scale measures a teacher's beliefs regarding actions in-class practice (Bandura, 1997). Therefore, it seems reasonable for teachers not to improve their self-efficacy through learning from Shanghai TIPs. Third, the negative coefficient (not statistically significant) appears because there is a large portion of non-education major teachers in the study sample. Ding (2010) explained that non-education major teachers might feel unconfident due to not having participated in college teacher preparation programs.

RQ2 indicated that, when teacher self-efficacy increases, first-year teachers in Shanghai public primary schools anticipate a reduction in teacher retention. This result is inconsistent with our hypothesis. One possible explanation for why teachers with high teacher self-efficacy would be less likely to stay in the teaching profession may be the imbalance between their “pay and expected gain.” Although first-year teachers feel confident as teachers, they may feel that the rewards (such as salary) do not meet their efforts and expectations, as well as their social status and social respect, even though the overall teaching profession is perceived as moderately prestigious in Shanghai. Wu (2018) reported that only 6% of Shanghai public primary school teachers reported being satisfied with their salaries. Thus, they may consider leaving the profession. From this author's perspective, first-year teachers might not have or barely have clear job prospects and career plans for their lifelong teaching career. Moreover, the majority of first-year teachers were 23–25 years old; thus, they may be able to be more flexible in their careers, allowing them to try out different jobs. Even though they possess teaching skills and strategies, they may not have considered the long-term effects of choosing teaching as a career.

Moreover, these data were collected in March of the school year, which is approximately two-thirds of the way through. It is time that first-year teachers slowly began to shift their teaching attitude from the disillusionment phase to the rejuvenation phase (Moir, 2011).

The disillusionment phase is a highly challenging phase for first-year teachers. They are overwhelmed with evaluations, teaching, having to cope with parents, and dealing with other school affairs. However, for the most part, they are uncertain about the process. This may lead to negative feelings such as anxiety, stress, and disenchantment. In the rejuvenation phase, first-year teachers slowly improve their teaching attitudes. However, this phase “tends to last into spring with many ups and downs along the way” (New Teacher Center, 2016, p. 3). Therefore, it is reasonable that first-year teachers, as the participants in this study, have a lower retention rate even though their teacher self-efficacy is relatively high. In addition, the school rank, district resources, and location may be considered factors in anticipated teacher retention. Teachers who work in schools with relatively low academic ranks and who are far from home or in rural areas may have additional reasons to consider leaving.

RQ 3 demonstrated that perceptions of TIP helpfulness were a statistically significant predictor of anticipated teacher retention. The helpfulness of TIPs accounted for nearly 8% of the additional variance in teacher self-efficacy, which is considered to be a medium effect. There are several possible reasons female teachers have relatively high-anticipated teacher retention compared to male teachers. The results are similar to those from Ding (2010) and Zhu's (2014) studies in China. Additionally, from the author's perspective, there are several reasons. At first, there were more female graduates majoring in education than male graduates in colleges. Zhu (2014) reported that the percentage of female college students in education in China is 65.3%, while only 34.7% are male students. The remarkable difference in gender among college graduates not only indicates that there are more women than men who choose to study education but also reveals that the expectations for women, more so than men, may, to some extent, include having a stable occupation such as teaching or accounting after graduation. In addition, men are expected to earn more than women. However, teaching in public primary schools may not pay as much as other positions. Thus, for some or all of these reasons, it seems reasonable that the retention rate of male teachers in public primary schools is lower than it is for female teachers.

Additionally, teachers who do not possess education majors are more likely to continue their teaching careers compared to those with education majors. As mentioned in the methodology, this study used dummy-coded college majors as a control variable. Non-education major teachers indicated that the participants' college majors were other than education, such as Chinese, English, mathematics, science, music, the arts, and others. Teachers who were not in education majors did not allow to attend teacher preparation programs in college. These results are consistent with those from previous studies suggesting that gender and college major affect teacher retention (Ding, 2010; Struyven and Vanthournout, 2014). In addition to the findings from previous studies, the author posited that the results might be related to the National Higher College Entrance Exam (NCEE), commonly known as “Gaokao,” in China. NCEE is an annual academic qualification test required of almost all high school graduates who wish to attend a university.

Zhang (2017) described the importance of the NCEE as “the pivotal moment for Chinese secondary students as their scores in large part determine their future—whether they can go to university, which institution they will be admitted to and, consequently, what careers await them” (para. 10). In other words, what major the candidate will learn in college is dependent on his or her NCEE score rather than his or her application. A candidate who is willing to obtain an education but who has not attained the minimum score required of education majors cannot be accepted as an education major in college. Therefore, it is reasonable that non-education major teachers may feel highly appreciative of the opportunity to enter and remain in the teaching profession.

RQ 4 asked, “Is there an indirect effect of the helpfulness of TIP on anticipated teacher retention *via* teacher self-efficacy?” The results provided evidence that teacher self-efficacy is not a mediator between the helpfulness of TIP and anticipated teacher retention among first-year teachers in Shanghai public primary schools. The primary reason teacher self-efficacy is not a mediator in the model is that there is no significant relationship between the helpfulness of teacher induction programs and teacher self-efficacy. The possible reasons for this have been discussed above (as part of the results for Research Question 1).

## 6. Limitations

Based on the threats to internal, external, construct, and statistical conclusion validity, as outlined in McMillan and Schumacher (2010), the following limitations of this study are acknowledged. First, this study relied on self-reported data from the participants, which may not accurately reflect their true feelings or actions. Second, the study used a convenience sample rather than a teacher database consisting of all Shanghai first-year teachers; therefore, the population's external validity was limited to those teachers with certain characteristics, such as those who responded. Third, the study investigated and had not anticipated actual first-year teacher retention. Therefore, some respondents may choose to remain despite their stated intentions. Finally, although we were careful in phrasing the research questions in terms of association rather than effects, a correlational design limited our ability to draw definitive conclusions. The results may be suggestive, but further research is needed to draw conclusions from the impacts TIPs have.

## 7. Suggestions for future research

This study's results indicate that the perception of Shanghai TIP helpfulness across all four components is positively related to overall anticipated teacher retention. To gain a deep understanding of the relationship between each type of Shanghai TIP component and anticipated teacher retention, additional analyses focused on separate components should be conducted. Moreover, qualitative research methods should be added. For example, a phenomenological study based on in-depth interviewing may reveal additional insights into how first-year teachers experience the TIP and, in particular, how those experiences may be linked with their sense of teaching efficacy and plan to remain a public primary school teacher in Shanghai.

Second, the study aimed to examine whether the perception of helpfulness regarding TIPs could increase teacher self-efficacy and, in turn, improve teacher retention rates. The study's results did not reveal that teacher self-efficacy is a mediator in this model. Therefore, future research is needed to propose and test other possible mediation pathways to explore potential indirect effects, as found in this study, in addition to what may be a direct effect.

Finally, it is interesting to note that teacher self-efficacy was statistically negatively correlated with anticipated teacher retention after controlling for gender, degree, and major. This suggests that first-year teachers in Shanghai public primary schools who possess higher levels of confidence in their abilities to engage students, manage the classroom, and implement effective instructional strategies are less likely to remain in their position. Therefore, future research is needed, including in-depth studies of first-year teachers with high levels of self-efficacy, which would enhance our understanding.

## Data availability statement

The original contributions presented in the study are included in the article/supplementary material, further inquiries can be directed to the corresponding author.

## Ethics statement

The studies involving human participants were reviewed and approved by University of the Pacific. The patients/participants provided their written informed consent to participate in this study.

## Author contributions

The author confirms being the sole contributor of this work and has approved it for publication.
